# Mitoxantrone Shows In Vitro, but Not In Vivo Antiviral Activity against Human Respiratory Syncytial Virus

**DOI:** 10.3390/biomedicines9091176

**Published:** 2021-09-07

**Authors:** Patricia G. de la Sota, Elena Lorente, Laura Notario, Carmen Mir, Oscar Zaragoza, Daniel López

**Affiliations:** Centro Nacional de Microbiología, Instituto de Salud Carlos III, 28220 Majadahonda, Spain; patric15@ucm.es (P.G.d.l.S.); elorente@isciii.es (E.L.); lnotario@externos.isciii.es (L.N.); carmen.mir@isciii.es (C.M.); ozaragoza@isciii.es (O.Z.)

**Keywords:** antivirals, bioluminescence, drugs, HRSV

## Abstract

Human respiratory syncytial virus (HRSV) is the most common cause of severe respiratory infections in infants and young children, often leading to hospitalization. In addition, this virus poses a serious health risk in immunocompromised individuals and the elderly. HRSV is also a major nosocomial hazard in healthcare service units for patients of all ages. Therefore, the development of antiviral treatments against HRSV is a global health priority. In this study, mitoxantrone, a synthetic anthraquinone with previously reported in vitro antiprotozoal and antiviral activities, inhibits HRSV replication in vitro, but not in vivo in a mice model. These results have implications for preclinical studies of some drug candidates.

## 1. Introduction

Human respiratory syncytial virus (HRSV) [1] is a member of the *Pneumoviridae* family of the Mononegavirales order. This enveloped Orthopneumovirus is the major cause of severe lower respiratory tract illnesses, such as bronchiolitis and pneumonia, in newborns and young children, with infection rates close to 70% in the first year of life [2]. At the age of 2–3 years, nearly all children have been infected by HRSV [2], and approximately 2–3% of infected infants must be hospitalized at higher rates than other respiratory viruses such as human metapneumovirus [3]. As the respiratory damage does not end with the resolution of the infection, some of these children will develop an increased risk for recurrent wheeze and asthma [4]. This virus causes repeated natural infections throughout life in people of all ages [5], although in healthy adults, mild infections are generally the most common clinical outcome, with a mortality risk comparable to that of influenza patients [6]. Generally, year-on-year fluctuations with a shift of dominance between the two viral antigenic subgroups described is produced in both children [7,8] and adults [9]. In addition, severe health risk is also relevant for the immunocompromised [10,11], elderly individuals [12,13], or pregnant women [14]. Worldwide, more than 3.4 million hospital admissions and approximately a quarter of a million deaths each year are associated with HRSV disease, mainly in developing countries [15,16]. Moreover, this virus is a major nosocomial hazard in hospital or healthcare service units for patients of all ages [17], involving an important medical as well as economic impact. After nearly 50 years of research, there are still no specific antiviral drugs and no licensed active vaccine against this relevant pathogen. Antiviral treatment development is among the priorities for different organizations such as the WHO and the PATH. In this study, mitoxantrone, a synthetic anthraquinone derivative chemical compound licensed as an anticancer drug [18], but with in vitro antiprotozoal and antiviral activities, was tested in vitro and in vivo as antiviral candidate against HRSV infection.

## 2. Materials and Methods

### 2.1. Mice, Cells, and Viruses

Rag2−/− BALB/c mice were bred and housed under specific pathogen-free conditions in the animal facilities of the “Instituto de Salud Carlos III” (ISCIII), Majadahonda, Madrid, Spain. All procedures involving animals and their care were approved by the ISCIII Ethics Committees and were conducted according to institutional guidelines. The human epithelial cell line HEp-2 was maintained in DMEM (Gibco BRL, Cheshire, UK) supplemented with 10% FBS, and cultured at 37 °C in a 5% CO_2_ atmosphere. Two recombinant HRSV viruses were utilized. First, the rrHRSV (kindly supplied by M.E. Peeples) [19]. This is a recombinant HRSV that contains the red fluorescent protein gene inserted as an extra gene immediately downstream of the viral promoter. The red fluorescent protein expression can be detected directly by FACS analyses of infected cells. Second, the rHRSV-Luc [20] with the firefly luciferase (*Luc*) gene inserted as an extra gene immediately downstream of the matrix gene. Replication of the *Luc*-encoding virus in living mice can be visualized by bioluminescent imaging [20].

### 2.2. In Vitro Infection of HEp-2 Cells and FACS Analysis

HEp-2 cells were incubated with rrHRSV at an MOI of 1 PFU/cell for 2 h at 37 °C to allow virus binding. A mock-infected control culture was included. The cells were further incubated for 24 h and then harvested for FACS analysis. Cellular DNA topoisomerase I (camptothecin, irinotecan, and topotecan) or II (mitoxantrone, doxorubicin, and etoposide) inhibitors and sulfaguanidine, utilized as negative control, were added 20 min prior to infection. No differences were found in the viability of the infected cells in the absence and presence of the different inhibitors. Data were acquired on a BD Accuri C6 FC flow cytometer (BD Biosciences, San Jose, CA, USA) and analyzed using BDAccuri Samples software (BD Bioscience). The percentage of inhibition is the mean of three independent experiments and was calculated as follows:

100 − 100 × (MFI ^HRSV with drug^ − MFI ^No HRSV^)/(MFI ^HRSV^ − MFI ^No HRSV^) (1)

To analyze the statistical significance of the assays, non-parametric Mann–Whitney U test was used. *p*-values < 0.05 were considered to be statistically significant.

### 2.3. Mouse Infections and In Vivo Luminescence Measurements

In the preclinical study carried out from January 2021 to May 2021, mice were anesthetized by a mixture of ketamine and xylazine (1 and 0.2 mg per mouse, respectively) and infected i.n. with 50 μL of PBS containing 5 × 10^4^ p.f.u. of rHRSV-Luc. For antiviral drug administration to animals, mitoxantrone was added to the drinking water of mice in order to have 5 mg/kg in the water consumed by the mice daily. This amount is the maximal tolerated dose previously described for mitoxantrone [21]. New preparations of drinking water were used every two days. Three control mice and three treated with mitoxantrone were anaesthetized and luminescence was measured 5 min after i.n. administration of 50 μL of PBS containing 0.75 mg kg^−1^ D-luciferin (Sigma, St. Louis, MO, USA). Photon emission of rHRSV-Luc-infected mice was measured using the IVIS-Lumina Serie III (Spectrum In Vivo Imaging System) imaging system (Xenogen Corp., Alameda, CA, USA). Living Image software (version 4.4, Caliper Life Sciences, Waltham, MA, USA) was used to measure the luciferase activities. Bioluminescence images were acquired for 1 min with f/stop = 1 and binning = 8. A digital false-colour photon emission image of the mouse was generated, and photons were counted within a constant region of interest corresponding to the surface of the chest encompassing the whole-airway area. Photon emission was measured as radiance in p s^−1^ cm^−2^ sr^−1^, as previously reported [20].

## 3. Results

### 3.1. In Vitro Anti-HRSV Activity of Mitoxantrone

**Figure 1:**
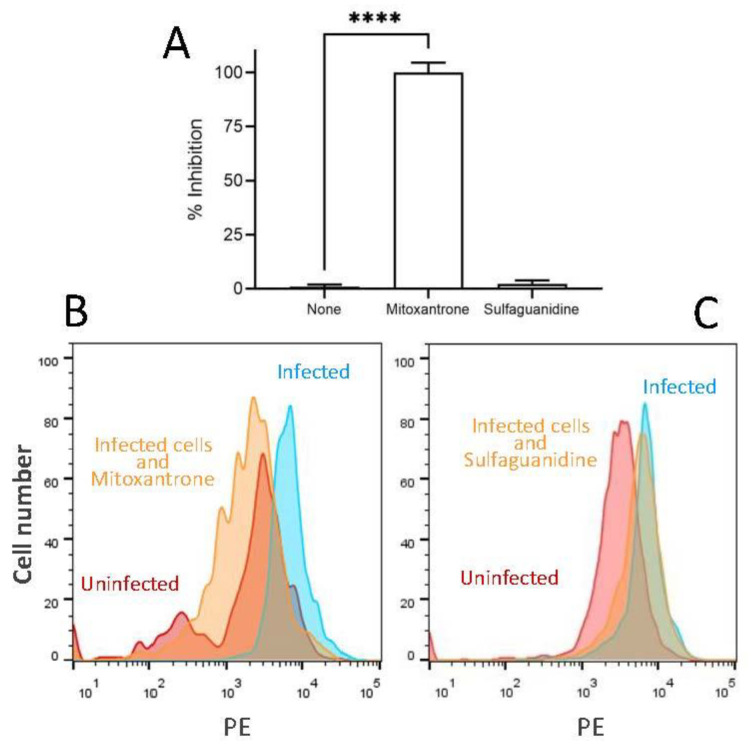
Inhibitory effect of mitoxantrone on HRSV infection of HEP-2 cells. (**A**) The expression of recombinant red protein in the rrHRSV-infected cells in the presence of different drugs was measured by flow cytometry. The results are calculated as the mean of three independent experiments ± SD. **** indicated *p*-value < 0.0001. Representative FACS experiments showing uninfected cells (red) and rrHRSV-infected cells untreated (blue) or treated with mitoxantrone or the irrelevant drug sulfaguanidine at 100 μM (yellow) are depicted in panels (**B**,**C**), respectively.

**Figure 2:**
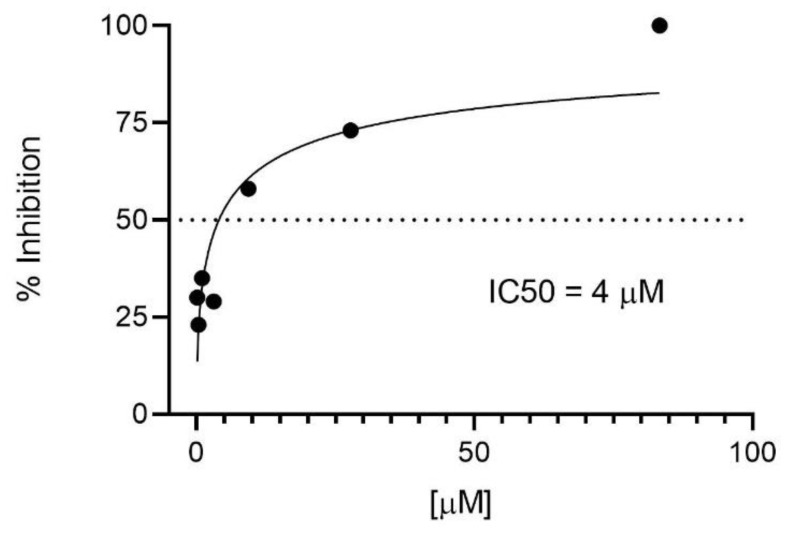
Inhibitory dose–response curve to determine the IC50 for mitoxantrone on HRSV infection of HEP-2 cells. The expression of recombinant red protein in the rrHRSV-infected cells was measured by flow cytometry as Figure 1. The concentration-dependent inhibitory dose-curve data were plotted as percentage of inhibition normalized to uninfected cell controls with applied curve fits calculated using GraphPad Prism.

The possible antiviral effect of the broad antipathogenic drug mitoxantrone against HRSV infection was carried out. Mitoxantrone, in contrast to the irrelevant drug sulfaguanidine, blocked HRSV replication measured by red protein expression of recombinant HRSV in infected cells (Figure 1). This inhibitory effect was dose-dependent with an IC_50_ = 4 μM (Figure 2). These results showed that mitoxantrone has antiviral activity against not only single-stranded, negative-sense RNA viruses such as HRSV.

### 3.2. DNA Topoisomerase I and II Inhibitors Do Not Block the In Vitro HRSV Replication

**Figure 3:**
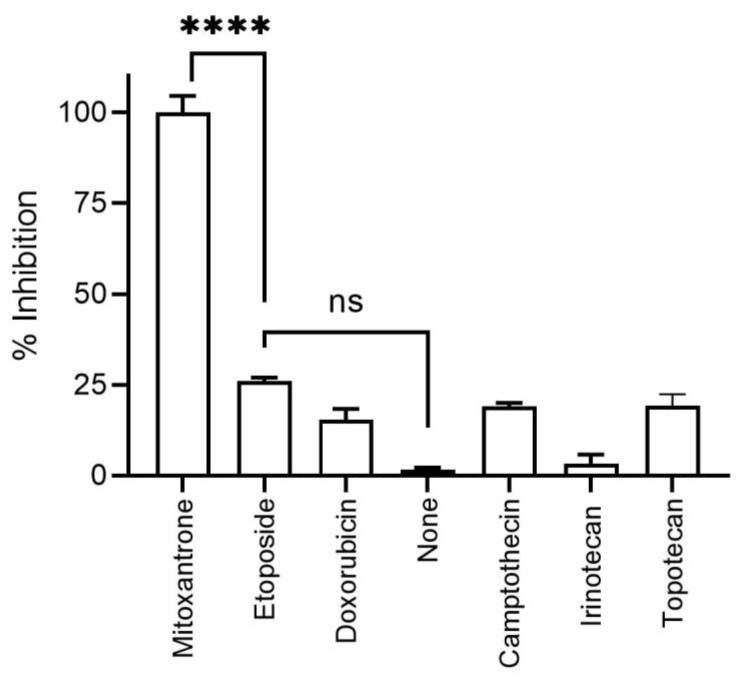
DNA topoisomerase inhibitors on HRSV infection of HEP-2 cells. The expression of recombinant red protein in the rrHRSV-infected cells in the presence of different DNA topoisomerase inhibitors was measured by flow cytometry as Figure 1. Mitoxantrone, doxorubicin, and etoposide are cellular DNA topoisomerase II inhibitors, whereas camptothecin, irinotecan, and topotecan are cellular DNA topoisomerase I inhibitors. The results are calculated as the mean of three independent experiments ± SD. **** indicated *p*-value < 0.0001.

Mitoxantrone is a cellular DNA topoisomerase II inhibitor. Therefore, other DNA topoisomerase II inhibitors such as doxorubicin and etoposide were evaluated against HRSV replication. Neither of these two, nor the other three DNA topoisomerase I inhibitors also analyzed, blocked HRSV replication (Figure 3).

### 3.3. Mitoxantrone Does Not Block the HRSV Replication in Immunodeficient Mice

**Figure 4:**
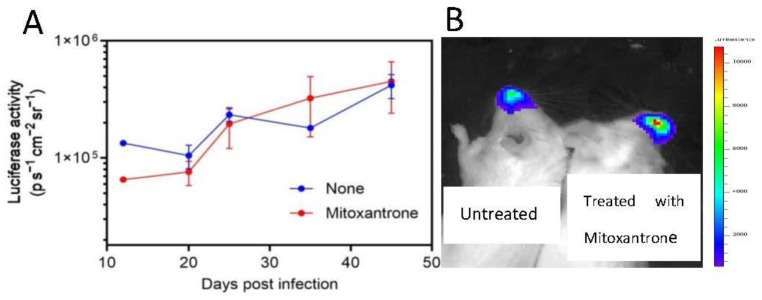
In vivo luminescence of rHRSV-Luc-infected mice treated with mitoxantrone. Panel (**A**) Six Rag2 mice were infected i.n. at day 0 by rHRSV-Luc. Bioluminescence of untreated (blue) or treated with mitoxantrone (red) mice was measured by capture of photon emission from the nose at different days post infection using the IVIS system. The scale on the left indicates the average radiance: the sum of the photons per second from each pixel inside the region of interest/number of pixels (ps^−1^ cm^−2^ sr^−1^). The results are calculated as the mean of three independent experiments ± SD. (**A**) Representative experiment is shown in panel (**B**).

The specific and strong effect of mitoxantrone on HRSV replication in cell culture prompted an evaluation of its efficacy in protecting mice against intranasal infection. Rag2 *knockout* *mice* present a homozygous disruption of the recombination activating gene 2. These animals exhibit total inability to initiate V(D)J rearrangement and fail to generate mature T or B lymphocytes. Thus, in these mice, the adaptive humoral and cellular immune responses are absent and they are unable to eliminate HRSV. Mice infected with rHRSV-Luc received doses of water placebo or water with mitoxantrone starting 5 days after virus infection, and in vivo bioluminescence intensity was determined. As shown in Figure 4, untreated animals developed a persistent infection. Similarly, the bioluminescence was not reduced for mice treated with mitoxantrone (Figure 4). Thus, the treatment with mitoxantrone had no benefit in the intranasal mouse infection model.

## 4. Discussion

In the long and expensive road towards identifying new drugs, the “new tricks for old drugs” strategy [22] can bypass the laborious and costly preclinical steps and go straight to clinical trials. This is especially relevant in the development of antivirals or antibiotics, where the interest of the pharmaceutical industry is very limited, and academic scientists can play a relevant role identifying new candidates to inhibitors. In this context, mitoxantrone, an anthracenedione with antineoplastic activity utilized mainly in acute lymphoblastic leukemia [23], breast cancer [24], and prostate cancer [25], is an interesting drug as it has previously been described as an antiprotozoal and antiviral agent. Mitoxantrone was described initially as a cellular DNA topoisomerase II inhibitor, which causes single- and double-stranded disruptions and DNA repair suppression by its intercalation between DNA bases. This mechanism of action would explain their antineoplastic and antiprotozoal activities. However, mitoxantrone also has in vitro antiviral activity against human herpes simplex virus by suppression of the viral immediate early genes, which are transcribed by cellular RNA polymerase II in a highly regulated cascade [26], although the direct interaction between this enzyme and the drug has not been described to date. In addition, mitoxantrone inhibits in vitro, but not in vivo vaccinia virus replication by blocking virion assembly, but not protein synthesis, by an unknown mechanism of action [21]. Recently, in vitro inhibition of SARS-CoV-2 has been described involving cell surface heparan sulfate as cofactor [27]. These pleiotropic effects, involving both different species and mechanisms of action, would suggest additional targets of this drug. Thus, in the current study, we tested the activity of mitoxantrone against HRSV replication. Our data showed that mitoxantrone (but not other cellular DNA topoisomerase I and II inhibitors) also prevents in vitro replication of HRSV. This fact is very interesting because HRSV is a single-stranded, negative-sense RNA virus, unrelated phylogenetically to poxviruses or herpesviruses, both double-stranded DNA pathogens that possesses large genomes or positive-sense single-stranded RNA coronaviruses. Thus, our data suggest that an additional mechanism of action to previously characterized or still unknown is involved in the anti-HRSV activity of mitoxantrone. In addition, all these data open the possibility that this drug may have additional antiviral properties against other viruses to those described by us and the other groups who have worked previously with mitoxantrone. This is a hypothesis that should be analyzed in depth in future studies.

In the next step of the “new tricks for old drugs” strategy, we analyzed the effect of mitoxantrone in an immunodeficient mouse model, which eliminates the contribution of the immune system to viral clearance, thus the real effect of the antiviral treatment is discovered. Unfortunately, as in many other drug candidates in multiple preclinical studies, mitoxantrone failed to control HRSV infection in the in vivo experiments. However, drug efficacy testing in mice has several limitations. First, the time of divergence between humans and rodents is estimated to be approximately 96 million years ago [28]. This implies that absorption, distribution, metabolism, excretion, effectiveness, and toxicity of drugs can be very different in organisms so phylogenetically distant. For example, corticosteroids are extensively teratogenic in animals, but not in humans [29], and thalidomide is not a teratogen in many animal species, but it is in humans [30]. In addition, humans and mice have different genes, regulatory regions, or even evolutionary paths. However, even though humans and mice share the same genes, and these are sufficiently conserved, the homolog genes can be used in different ways in each specie. Thus, as “mice are not simply furry little people”, animal studies are often poor predictors of human reactions. In this context, a chemical compound such as mitoxantrone with such broad antiprotozoal and antiviral activities could be directly analyzed in humans despite the failure of in vivo animal testing against HRSV and vaccinia virus [21].

## Data Availability

The data presented in this study are available on request from the corresponding author.
